# Inhibition of FLT3 Expression by Green Tea Catechins in FLT3 Mutated-AML Cells

**DOI:** 10.1371/journal.pone.0066378

**Published:** 2013-06-20

**Authors:** Bui Thi Kim Ly, Hoang Thanh Chi, Makoto Yamagishi, Yasuhiko Kano, Yukihiko Hara, Kazumi Nakano, Yuko Sato, Toshiki Watanabe

**Affiliations:** 1 Department of Medical Genome Sciences, Graduate School of Frontier Sciences, University of Tokyo, Tokyo, Japan; 2 Division of Hematology, Tochigi Cancer Center, Utsunomiya, Tochigi, Japan; 3 Tea Solution, Hara Office Inc., Sumida-ku, Tokyo, Japan; 4 The Japanese Red Cross College of Nursing, Shibuya-ku, Tokyo, Japan; Weizmann Institute of Science, Israel

## Abstract

Acute myeloid leukemia (AML) is a heterogeneous disease characterized by a block in differentiation and uncontrolled proliferation. *FLT3* is a commonly mutated gene found in AML patients. In clinical trials, the presence of a FLT3-ITD mutation significantly correlates with an increased risk of relapse and dismal overall survival. Therefore, activated FLT3 is a promising molecular target for AML therapies. In this study, we have shown that green tea polyphenols including (−)-epigallocatechin-3-gallate (EGCG), (−)-epigallocatechin (EGC), and (−)-epicatechin-3-gallate (ECG) suppress the proliferation of AML cells. Interestingly, EGCG, EGC and ECG showed the inhibition of FLT3 expression in cell lines harboring FLT3 mutations. In the THP-1 cells harboring FLT3 wild-type, EGCG showed the suppression of cell proliferation but did not suppress the expression of FLT3 even at the concentration that suppress 100% cell proliferation. Moreover, EGCG-, EGC-and ECG-treated cells showed the suppression of MAPK, AKT and STAT5 phosphorylation. Altogether, we suggest that green tea polyphenols could serve as reagents for treatment or prevention of leukemia harboring FLT3 mutations.

## Introduction

Acute myeloid leukemia (AML) is the most common type of adult leukemia, affecting mostly elder people and its incidence increases with the age. It is an aggressive disease that involves rapid growth of abnormal leukemic cells in the bone marrow, resulting in failure of production of normal blood cells [1]. *FLT3* (*Fms-like tyrosine kinase 3*) is a commonly mutated gene found in AML patients with the approximately 5–30% of the cases of AML [2]. FLT3 is one of the members of the subclass III of Receptor Tyrosine Kinase (RTK) family and is normally expressed on the cell surface of hematopoietic progenitor cells but expression is lost upon cell maturation [3]. A very high expression of FLT3 has been found in around 70–100% AML patients that usually associate with a worse prognosis [4], [5].

*FLT3* mutations are predominantly detected in juxtamembrane domain (JMD) [6] and in tyrosine kinase domains (TKD) including the in-frame internal tandem duplication (*FLT3*-ITDs) [6], the point mutation [2], [7] and the insertion of six bases [8]. *FLT3*-ITDs have been found mostly in JMD [6] and few cases in TKD [9] with the approximation of 15–35% of AML [2], [10], [11], [12], whereas *FLT3* point mutations was predominantly found in activation loop of TKD (at D835 [7] and I836 position [2]) but rare in JMD [13] with approximately 5–10% of AML patients [2], [10], [11], [12]. The last mutation form was the insertion of a glycine and a serine between amino acids 840 and 841 of *FLT3* [8]. *FLT3* mutations result in a ligand-independent receptor dimerization, phosphorylation and constitutive activation of downstream signaling molecules including the RAS/RAF/MEK/ERK kinases, PI3-kinase and STAT5 kinases [14], [15], [16], [17]. In clinical, the presence of a FLT3-ITD mutation significantly correlates with an increased risk of relapse and dismal overall survival with the median survival after the first relapse has been reported to be ≤5 months [18], [19]. Therefore, activated FLT3 is a promising molecular target for AML therapies.

Currently, several small molecule FLT3-tyrosine kinase inhibitors (FLT3-TKIs) have been developed and examined in AML patients as single agents or in combination with chemotherapy. Up to now, six oral FLT3 inhibitors, including CEP-701, PKC412, BAY 43-9006, SU11248, MLN-518 and KW-2449, the i.v. compound SU5416 and AC220 have been investigated as monotherapy in clinical trials. In addition, FLT3-directed antibody therapy (IMC-EB10) is currently being investigated in a phase 1 clinical trial. FLT3-TKI monotherapy has been proven to efficiently target FLT3-mutated AML blasts [20]. However, approval of these agents for FLT3-associated diseases is still challenging, which was suspected to be due to the failure to fully inhibit FLT3 in tumors and undesirable drug properties [21].

In this study, we evaluated the anti-cancer effect of green tea polyphenols including (−)-epigallocatechin-3-gallate (EGCG), (−)-epigallocatechin (EGC), (−)-epicatechin-3-gallate (ECG), and (+)-Catechin (C) in a group of AML cell lines harboring FLT3 mutation. It is well documented that polyphenols of green tea show anti-cancer effects on many types of human malignancies, but not to their normal counterpart [22]. Currently, green tea is now developing as a cancer preventive drug in the USA and Europe [23], [24]. Our results show that EGCG, EGC and ECG treatment disrupts the association of Hsp90 with FLT3-ITD and results in reduced levels of FLT3 expression in AML harboring mutated FLT3.

## Materials and Methods

### 2.1. Cell Lines, Culture Conditions

Experiments were conducted using four human leukemia cell lines: two sister cell lines MOLM-13 and MOLM-14 that were established from a patient with acute monocytic leukemia (M5a) harboring t(9;11) [25]; MV4-11 from a patient with AML carrying t(4;11) [26] and KOCL-48 from an infant leukemic patient carrying t(4;11) [27].

In MOLM-13 and MOLM-14 cells, two mutations within *FLT3* exon 14 were detected: ITD of 21 bps corresponding to codons Phe594-Asp600 and a novel missense nucleotide substitution at the codon 599 (Tyr599Phe) [28], [29]. Two kinds of mutations were located on the same allele [29]. In MV4-11 cells, there are an ITDs of 30 bps within *FLT3* exon 14 corresponding to codons Tyr591-Asp600, and a Tyr591His mutation [28], [29]. In KOCL-48 cell line, only *FLT3*-Asp835Glu mutation was detected [28].

The cell line THP-1 came from the peripheral blood of a one-year old infant male with monocytic AML [30]. They do not contain a known FLT3 mutation and have high endogenous wild-type FLT3 (FLT3-WT) expression. THP-1 cells will be used as a negative control in this study.

The cells were grown in RPMI 1640 medium (Sigma-Aldrich, Japan K.K., Tokyo, Japan) supplemented with 10% heat-inactivated fetal bovine serum (FBS) (JRH Biosciences, Lenexa, KS, USA), 100 IU/ml penicillin, and 0.1 mg/ml streptomycin (Nakalai Tesque, Kyoto, Japan) in a humidified incubator of 5% CO_2_ at 37°C.

### 2.2. Reagents

Reagents were obtained as follows: EGCG, EGC, ECG, and C (purified powder) were generously gifted by one of us Dr. Yukihiko Hara (Japan), PKC412 was purchased from Sigma-Aldrich Japan K.K. (Tokyo, Japan) and 17-allylamino-17-desmethoxygeldanamycin (17-AAG) was purchased from Calbiochem (Darmstadt, Germany). All reagents were dissolved in dimethylsulfoxide (DMSO) (Wako Pure. Chemical Industries, Osaka, Japan). Controlled cells were cultured with the same concentration of carrier DMSO as used in the highest dose of reagents. The concentration of DMSO was kept under 0.1% throughout all the experiments to avoid its cytotoxicity.

### 2.3. Cell Proliferation Assays

Cell proliferation was determined by trypan blue dye exclusion test as described previously [31]. Briefly, cells were seeded in 6-well plates at a density of 1×10^5^ cells/ml in the presence of different concentrations of EGCG, EGC, ECG and C for 72 hours. After the treatment, 10 µl of the cell suspension was mixed with 10 µl of 0.4% trypan blue, and alive cells were counted manually using a hemacytometer. Results were calculated as the percentage of the values measured when cells were grown in the absence of reagents.

### 2.4. Western Blot Analysis

Cells were plated onto 10 cm dishes at a density of 1×10^5^ cells/ml in the presence of various concentrations of reagents. After incubation for indicated durations, cells were collected and washed twice with PBS (−). Cells were then dissolved in a protein lysis buffer containing 5 mM EDTA, 50 mM NaF, 10 mM Na_2_H_2_P_2_O_7_, 0.01% Triton X-100, 5 mM HEPES, 150 mM NaCl, 1 mM Na_3_VO_4_, 1 mM phenylmethylsulfonyl fluoride, and 75 µg/mL aprotinin on ice for 30 min with brief vortex of 4 times with every 10 min. After centrifugation at 13,000 rpm at 4°C for 10 min, total cell lysates were collected. Protein samples were electrophoresed through a polyacrylamide gel and transferred to a Hypond-P membrane (Amersham, Buckinghamshire, UK) by electro-blotting. After washing, the membrane was probed with antibodies and antibody-binding was detected using BCIP/NBT substrate (Promega). The following antibodies were obtained from Santa Cruz Biotechnology (Santa Cruz, CA, USA): FLT-3/FLK-2 (S-18) (sc-480), STAT5 (C-17) (sc-835) and survivin (sc-17779). Anti-actin (A2066) was from Sigma-Aldrich. p44/42 MAPK (Erk1/2), phospho-p44/42 MAPK (Thr202/Tyr204), AKT, phospho-AKT (Ser473), phospho-STAT5 (Tyr694), caspase-3, caspase-9 (C9) and XIAP antibodies were from Cell Signaling Technology Japan (Tokyo, Japan). Anti-PARP antibody was from WAKO Chemicals (Osaka, Japan).

### 2.5. Co-immunoprecipitation

For immunoprecipitation (IP), MOLM-13 cells were treated with EGCG, EGC or ECG for 8 hours and then harvested. Cells were lysed as indicated above. Then, 500 µg of protein total cell lysates were immunoprecipitated with anti-Hsp90 (Santa Cruz, CA, USA) overnight at 4°C. Protein G Sepharose 4 Fast Flow (Amersham Pharmacia Biosciences, Tokyo, Japan) was then added for 2 hours. The immunoprecipitates were washed three times with Tris buffered saline-Tween. The bound proteins were resolved by SDS-PAGE and analyzed by Western blotting.

### 2.6. Determination of Apoptosis

MOLM-14 cells were treated with EGCG, EGC or ECG for 16 hours. The apoptotic cell was evaluated by PE Annexin V (BD PharMingen) and analyzed by FACS Calibur (Becton, Dickinson). Collected data were analyzed by FlowJo software (Tree Star).

### 2.7. Isobologram

The dose-response interactions between EGCG and PKC412 on the MOLM-13, MOLM-14, MV4-11 and KOCL-48 cells were evaluated at the IC_50_ level by the isobologram of Steel and Peckham [32]. The IC_50_ was defined as the concentration of the reagent that produced 50% cell growth inhibition. The concept of the isobologram has been described in detail elsewhere [32]. We used this isobologram because this method can cope with any agents with unclear cytotoxic mechanisms and a variety of dose-response curves of anticancer agents [32].

### 2.8. Statistical Analysis

Data for isobologram were analyzed as described elsewhere [33]. When the observed data points of the combinations mainly fell in the area of supraadditivity or in the areas of subadditivity and protection, i.e., the mean value of the observed data was smaller than that of the predicted minimum values or larger than that of the predicted maximum values, the combinations were considered to have a synergistic or antagonistic effect, respectively. To determine whether the condition of synergism (or antagonism) truly existed, a statistical analysis was performed. The Wilcoxon signed-ranks test was used for comparing the observed data with the predicted minimum (or maximum) values for additive effects, which were closest to the observed data. Probability (P) values <0.05 were considered significant. Combinations with P≥0.05 were regarded as indicating additive to synergistic (or additive to antagonistic) effects. The other data were analyzed by Student’s t test.

## Results

### 3.1. Growth-inhibitory Effect of EGCG, EGC, ECG and C on FLT3 Mutated-AML Cells

**Figure 1:**
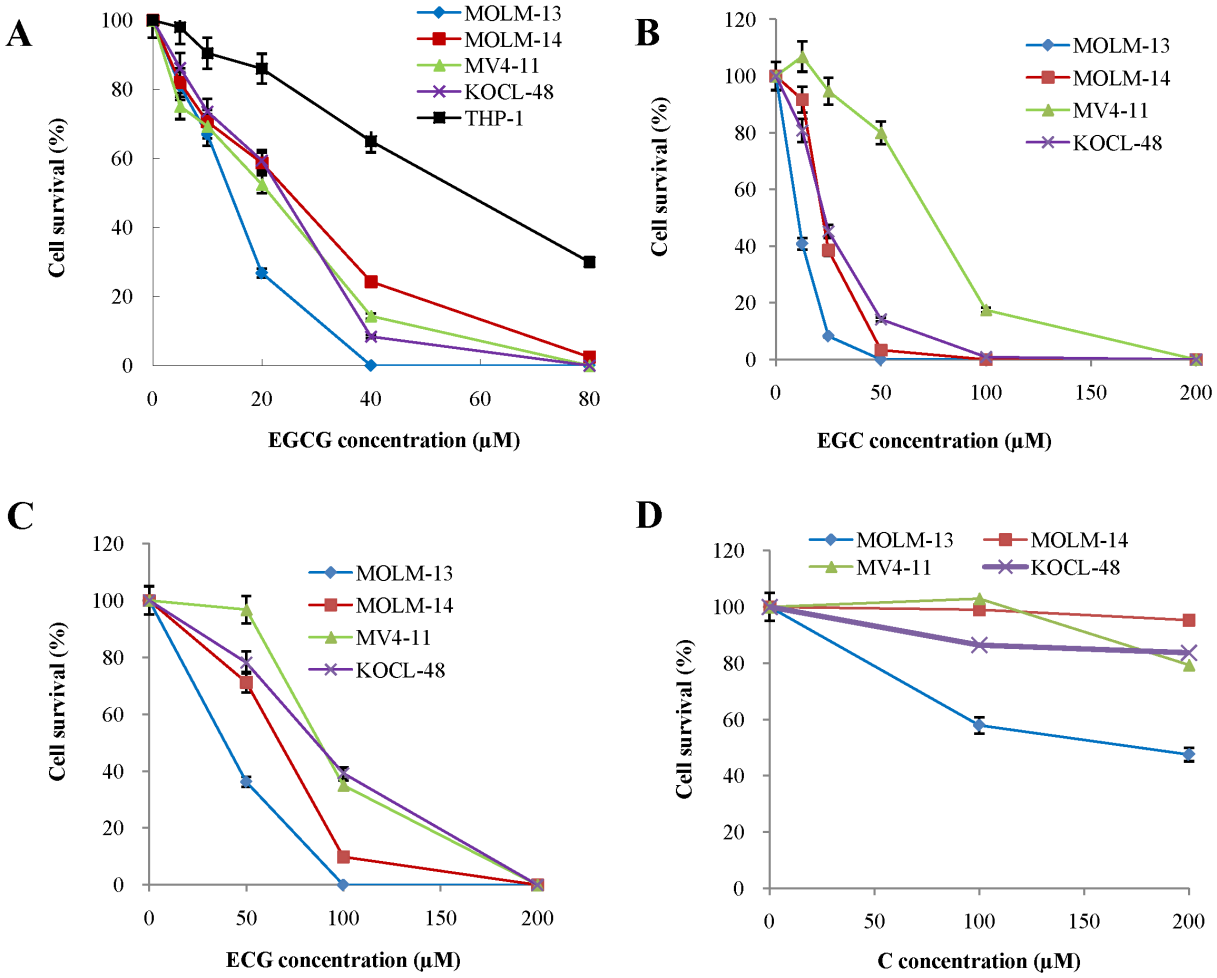
Effect of EGCG, EGC, ECG and C on cell proliferation of MOLM-13, MOLM-14, MV4-11 and KOCL-48 cell lines. MOLM-13, MOLM-14, MV4-11, KOCL-48 and THP-1 cells at a density of 1×10^5^ cells/ml were treated with indicated concentration of EGCG, EGC, ECG, C or DMSO alone as control for 72 hours. The number of alive cells was counted after trypan blue exclusion test. Results were calculated as the percentage of the control values.

To test the inhibitory effect of EGCG, EGC, ECG and C on the growth of AML cell lines, MOLM13, MOLM14, MV4-11 and KOCL-48 cells were incubated either with the carrier DMSO alone (control) or with different concentrations of reagents for 72 hours. Cell proliferations were evaluated using the trypan blue exclusion test. The result showed that EGCG, EGC and ECG significantly inhibited the cell proliferation of MOLM-13, MOLM-14, MV4-11 and KOCL-48 cells in a dose-dependent manner (Fig. 1 A, B and C). Whereas, the growth-inhibition effect of (+)-Catechin on these cells was less sensitive than others (Figure 1D). Altogether, green tea polyphenols showed the anti-proliferation effects on AML cells.

### 3.2. Down-regulation of FLT3 Expression and its Downstream Molecules in EGCG-Treated AML Cells

**Figure 2:**
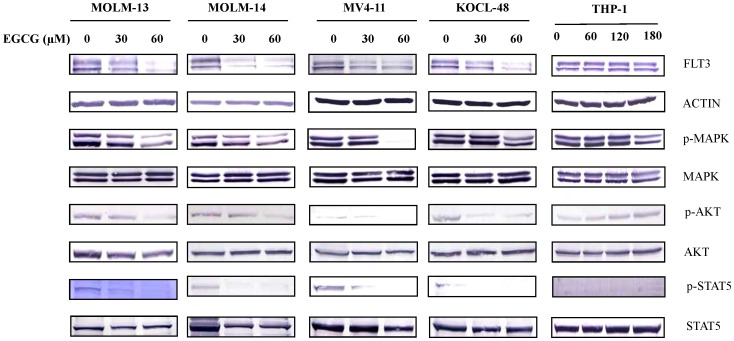
Down-regulation of FLT3 expression and its downstream molecules in EGCG-treated AML cells. MOLM-13, MOLM-14, MV4-11, KOCL-48 and THP-1 cells at a density of 1×10^5^ cells/ml were treated with indicated concentration of EGCG or DMSO alone as control for 8 hours. Total cell lysates were subjected to western blot analysis with indicated antibodies.

To address the mechanism of the EGCG-mediated growth inhibition in *FLT3* mutated-AML cells, we analyzed the expression of FLT3 protein in these cells treated with or without EGCG by western blotting. Interestingly, the expression level of FLT3 protein was significantly decreased after 8 hours exposure of MOLM-13, MOLM-14, MV4-11 and KOCL-48 cells (FLT3 mutated cells) to different concentrations of EGCG (Fig. 2, the first row). However, In THP-1 cells (FLT3-WT cells), the level of FLT3 expression did not change even at high EGCG concentration treatment (180 µM) (Fig. 2, the first row). The data suggested that EGCG specifically targets mutant FLT3 rather than wild-type FLT3.

We reasoned that the down-regulation of FLT3 expression would lead to inhibition of its activity, subsequently suppress the activity of its down-stream molecules. To make it clear, we measured the activity of MAPK, AKT and STAT5 in MOLM-13, MOLM-14, MV4-11, KOCL-48 and THP-1 cells treated with or without EGCG. The inhibitions of MAPK, AKT and STAT5 activity (p-MARK, p-AKT and p-STAT5) were observed in MOLM-13, MOLM-14, MV4-11 and KOCL-48 cells after 8 hours incubation with EGCG (Fig. 2). Although EGCG caused the suppression of cell growth in THP-1 cells (IC_50_ ≈ 60 µM EGCG, Fig. 1A), only activity of MAPK but not AKT and STAT5 was decreased at very high concentration of EGCG (180 µM) (Fig. 2).

### 3.3. Down-regulation of FLT3 Expression and its Downstream Molecules in EGC- and ECG-treated AML Cells

**Figure 3:**
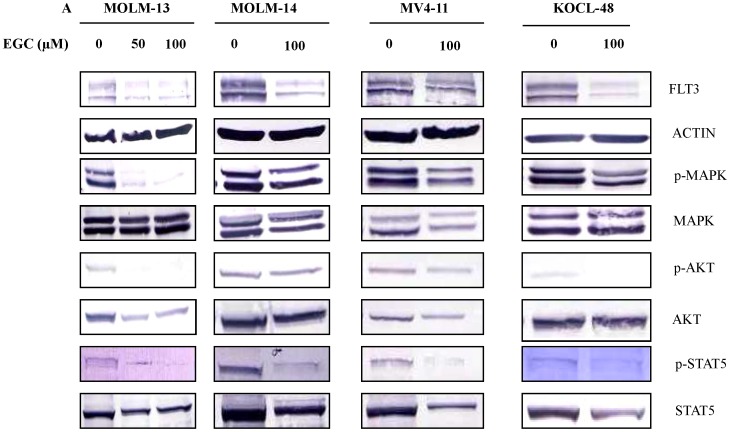
Down-regulation of FLT3 expression and its downstream molecules in EGC-treated AML cells. MOLM-13, MOLM-14, MV4-11 and KOCL-48 cells at a density of 1×10^5^ cells/ml were treated with indicated concentration of EGC or DMSO alone as control for 8 hours. Total cell lysates were subjected to western blot analysis with indicated antibodies.

**Figure 4:**
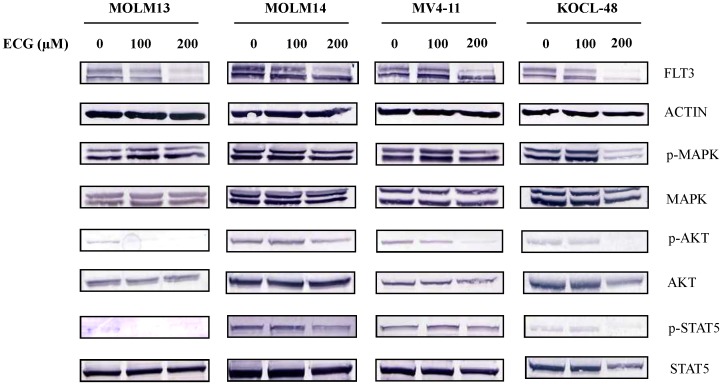
Down-regulation of FLT3 expression and its downstream molecules in ECG-treated AML cells. MOLM-13, MOLM-14, MV4-11 and KOCL-48 cells at a density of 1×10^5^ cells/ml were treated with indicated concentration of ECG or DMSO alone as control for 8 hours. Total cell lysates were subjected to western blot analysis with indicated antibodies.

To check whether EGC and ECG suppress the expression of FLT3, MOLM-13, MOLM-14, MV4-11 and KOCL-48 cells were treated with 100 µM EGC or 100 or 200 µM ECG for 8 hours. Western blot was performed to analyze the expression of FLT3. EGC-treated cells showed not only the suppression of FLT3, but also the suppression of phosphorylation of MAPK, AKT and STAT5 (Fig. 3). Similarly, the suppression of FLT3 expression and phosphorylation of its downstream molecules were also observed in ECG-treated cells (Fig. 4).

### 3.4. EGCG, EGC and ECG Suppressed FLT3 Expression through Hsp90

**Figure 5:**
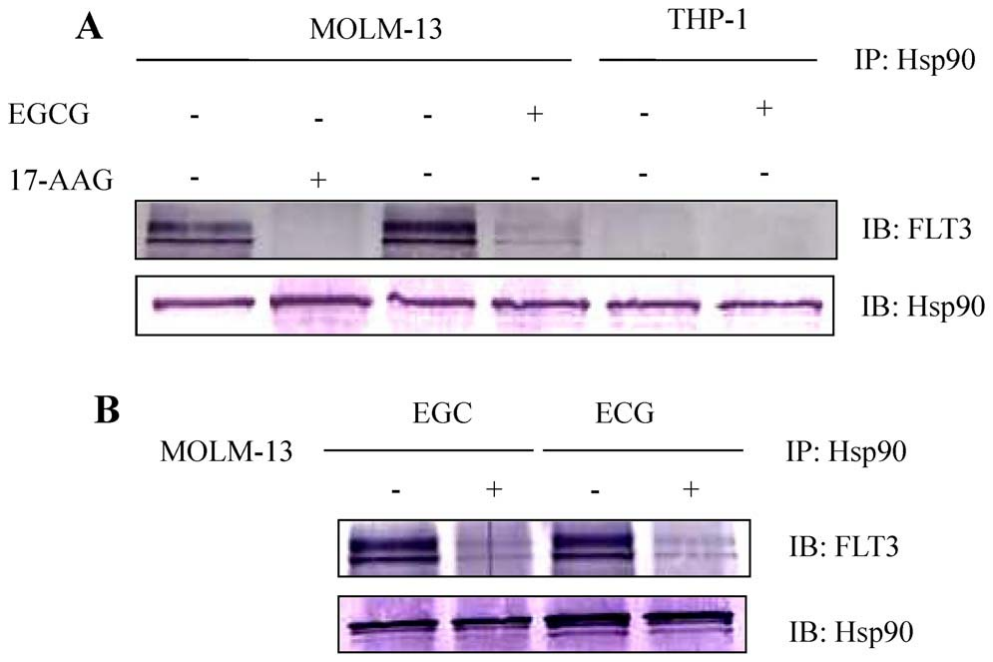
EGCG, EGC and ECG suppressed FLT3 expression through Hsp90. MOLM-13 cells at a density of 1×10^5^ cells/ml were treated with 60 µM EGCG (A, lane 4), 100 µM EGC, 200 µM ECG (B, lane 2, 4) or DMSO alone as control for 8 hours. Total cell lysates were immunoprecipitated with anti-Hsp90. Precipitated protein were subjected to western blot analysis with anti-FLT3 and anti Hsp90. MOLM-13 cells treated with 2 µM 17-AAG was used as control (A, lane 2).

EGCG has been demonstrated to be an inhibitor of Hsp-90 [34]. Moreover, recently FLT3-ITD but not FLT3-WT has been shown as a client protein of Hsp-90 [35], [36]. Thus it could be assumed that mutant FLT3 expression is suppressed by EGCG treatment might through inhibition of Hsp-90. To confirm this hypothesis, total cell lysates of MOLM-13 and THP-1 cells after EGCG treatment were collected and immunoprecipitated with indicated anti-body as shown in Figure 5A. The results of immunoprecipitation showed that only FLT3-ITD could physically interact with Hsp-90 but not FLT-WT (Fig. 5A lane 3 and 5). In addition, EGCG treatment disrupts the association of Hsp90 with FLT3-ITD and results in reduced levels of FLT3 expression (Fig. 5A lane 4). Similar results are observed in EGC-, and ECG-treated MOLM-13 cells (Fig. 5B) suggesting that mechanism of EGC and ECG on suppression of FLT3 expression might through inhibit the activity of Hsp-90.

### 3.5. EGCG, EGC and ECG Induced Apoptosis in FLT3 Mutated-AML Cells

**Figure 6:**
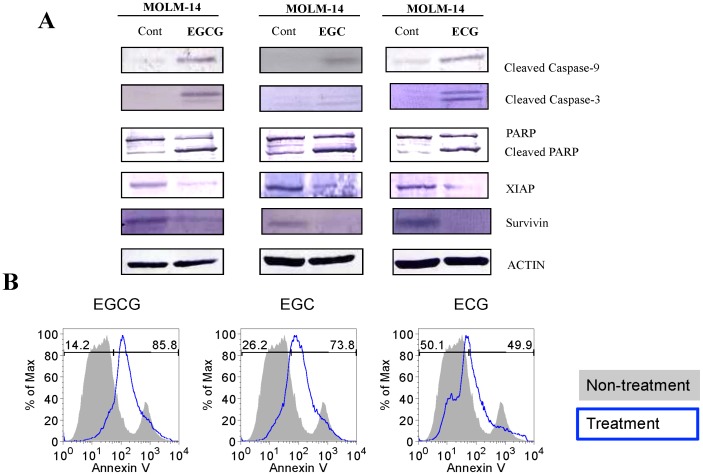
EGCG, EGC and ECG induced apoptosis in MOLM-14 cells. MOLM-14 cells at a density of 1×10^5^ cells/ml were treated with 60 µM EGCG, 100 µM EGC, 200 µM ECG or DMSO alone as control for 16 hours. Total cell lysates were subjected to western blot analysis with indicated antibodies (A) or staining with PE-Annexin V and analyzed by FACS Calibur. Collected data were analyzed by FlowJo software (B).

Next, we demonstrated that EGCG, EGC and ECG induced apoptosis in FLT3 mutated cell lines. We have checked the appearance of some apoptotic markers in MOLM-14 cells after EGCG, EGC or ECG treatment by western blot. The bands of cleaved-caspase-9, cleaved-caspase-3 and cleaved-PARP were observed after 8 hours incubated with EGCG, EGC or ECG (Fig. 6A). Moreover, EGCG, EGC or ECG treatment showed the inhibition of the expression of anti-apoptotic molecules such as survivin and XIAP in MOLM-14 cells (Fig. 6A).

The results from PE-Annexin V staining that shown in figure 6B indicated that EGCG, EGC and ECG induce apoptosis in MOLM-14 cells treated with EGCG, EGC or ECG. Overall, the EGCG-, EGC- or ECG- induced cell death in MOLM-14 is apoptosis.

### 3.6. Cytotoxic Effects of EGCG in Combination with PKC412

**Figure 7:**
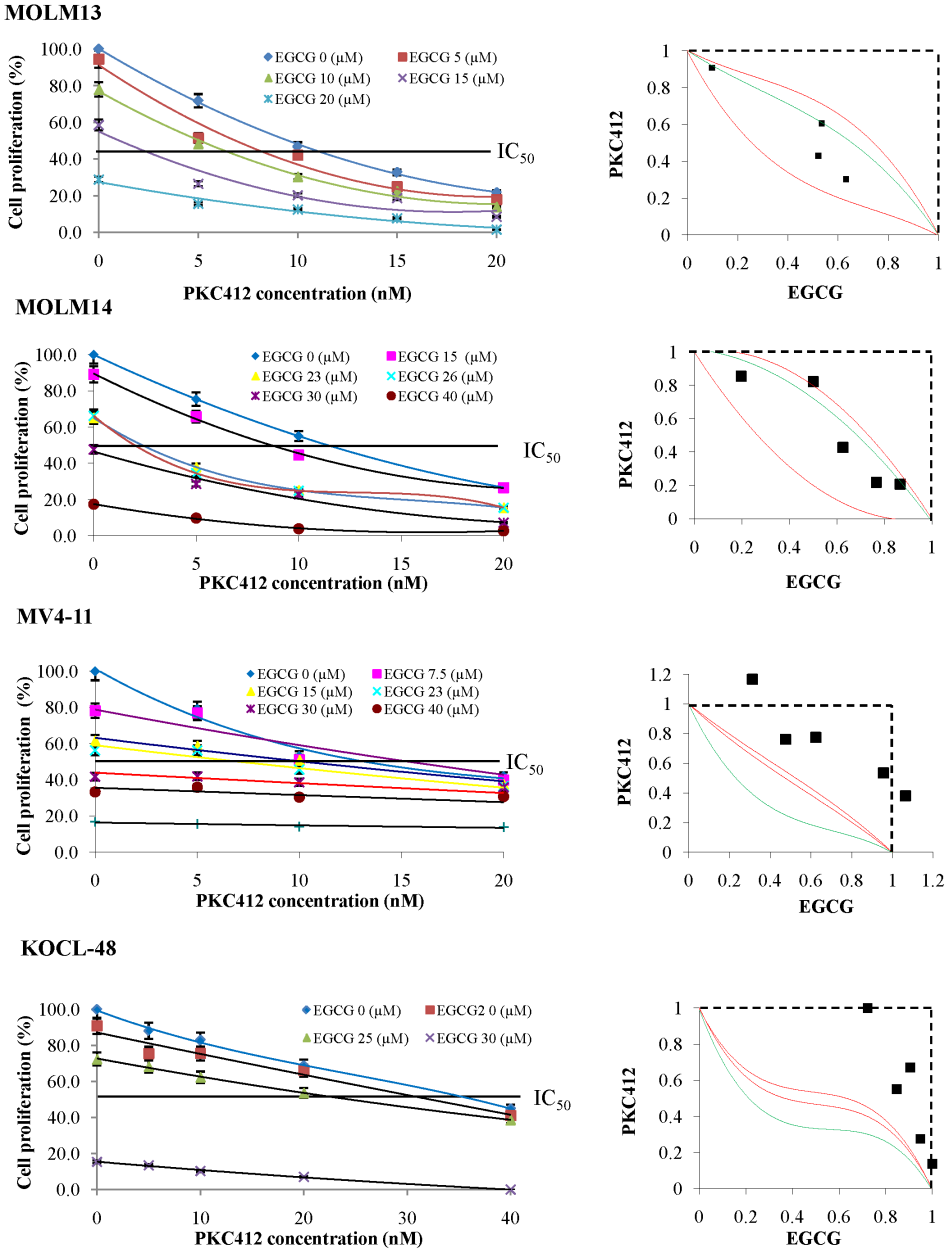
Isobolograms of simultaneous exposure to EGCG and PKC412 in MOLM-13, MOLM-14, MV4-11 and KOCL-48 cell lines. The isobolograms shown are representative of at least three independent experiments. Each point represents the mean value of at least three independent experiments. The combination of EGCG with PKC412 showed additive effect (MOLM-13 and MOLM-14) and antagonism effect (MV4-11 and KOCL-48).

EGCG is one of the most studied polyphenol of green tea and shows the strongest anti-cancer effect compared to other polyphenols of green tea (Fig. 1). In this study, we evaluated the combination effect of EGCG and PKC412. Interestingly, the results showed that the combination of EGCG and PKC412 treatment strongly suppressed cell growth in MOLM-13, KOCL-48, MV4-11 and MOLM-14 cells compared to EGCG or PKC412 treatment (Fig. 7). However, the combined effects of simultaneous exposure to these drugs differed among MOLM-13, MOLM-14 and KOCL-48, MV4-11 cell lines. In MOLM-13 and MOLM-14 cells, the combined data points fell within the envelope of additivity. The mean value of the data (0.423 and 0.414 respectively) was larger than that of the predicted minimum data (0.379 and 0.121 respectively) and smaller than that of the predicted maximum data for an additive effect (0.766 and 0.549 respectively; Table 1), indicating that simultaneous exposure to EGCG and PKC412 produced an additive effect. In KOCL-48 and MV4-11 cells, the combined data points fell mainly in the area of subadditivity, and the mean values of the observed data (0.887 and 0.803 respectively) were larger than those of the predicted maximum additive data (0.410 and 0.579 respectively; Table 1) which were regarded as antagonism effect.

**Table 1:** Mean values of observed data and predicted minimum and maximum values of the combination of EGCG and PKC412.

Cell lines	n	Observed data	Predicted values for an additive effect		Effect
			Minimum	Maximum	
MOLM-13	4	0.423	0.379	0.766	Additive
MOLM-14	5	0.414	0.121	0.549	Additive
MV4-11	5	0.803	0.205	0.410	Antagonism (<0.01)
KOCL-48	5	0.887	0.467	0.579	Antagonism (<0.01)

## Discussion

It is well documented that polyphenols of green tea, used as a beverage for over 5,000 years, have anti-cancer effects on many types of human malignancies, but not to their normal counterpart [22]. So far, we have demonstrated that EGCG suppressed the cell proliferation and caused apoptotic cell death in GIST cells by inhibition of KIT activity [31]. In this work, we have reported that polyphenols of green tea caused apoptotic cell death in *FLT3*-mutated cell lines (Fig. 6) by suppressing the expression of FLT3 and ultimately suppressing the activity of AKT, MAPK and STAT5 (Fig. 2, 3 and 4).

Recent studies indicated that RTKs are one of the critical targets of EGCG to inhibit cancer cell growth. The previous studies provided evidences that EGCG inhibited the activation or expression of some RTKs including epidermal growth factor receptor (EGFR) [37], human epidermal growth factor receptor 2 (HER2), HER3 [38], [39], vascular endothelial growth factor receptor (VEGFR) [40], platelet derived growth factor receptor (PDGFR) [41], fibroblast growth factor receptors (FGFR) [37], insulin-like growth factor 1 receptor (IGF-1R) [42], [43] and KIT [31]. In this report, we showed that EGCG, EGC and ECG suppressed the FLT3 expression (Fig. 2, 3 and 4) in cell lines harboring FLT3 mutation but not in THP-1 cells that carrying FLT3 wild-type.

One of the hypothesizes that can be explains for the difference effect of EGCG on FLT3 WT and FLT3 mutant expression would be through the mediator heat shock protein 90 (Hsp90). Hsp90, a highly abundant molecular chaperone in the stress response, assists maturation of more than 200 proteins, which include transmembrane tyrosine kinases (Her-2, EGFR), metastable signaling proteins (Akt, K-ras, Raf-1), mutated signaling proteins (p53, v-Src), chimeric signaling proteins (Bcr-Abl), cell cycle regulators (Cdk4, Cdk6), and steroid receptors (androgen, estrogen, and progesterone receptors) [44], [45], [46], [47], [48]. Both the N- and C-terminal domain of Hsp90 contains specific ATP binding pockets [49], [50]. Hsp90 dimerizes through its C-terminal end. Upon ATP binding, the two N-termini of the dimer associate to form a molecular “clamp” with a client protein to exert its chaperone function [51], [52]. Previous studies show that FLT3-ITD but not FLT3-WT is a client protein of Hsp90 in murine tranfected 32D cell lines [35] and primary AML cells [36].

Recently, EGCG has been demonstrated as inhibitor of Hsp90. EGCG acts by binding at or near a C-terminal ATP binding site to inhibit dimerization and promote an Hsp90 conformation that interferes with its chaperone activity for a client protein [34]. In the same year, another group also reported that EGCG inhibits Hsp90 function by impairing Hsp90 association with cochaperones including Hsc70 and p23 in pancreatic cancer cell line Mia Paca-2 [53]. In 2010, Tran et al. reported that in MCF-7 human breast cancer cells, EGCG specifically inhibited the expression of Hsp90 by inhibiting the promoter activity of Hsp90. However, in this study, we did not observed the decrease of Hsp90 expression after 8 hours treated MOLM-13, MOLM-14, MV4-11, KOCL-48 and THP-1 cells with 60 µM EGCG (data not shown). We hypothesized that the suppression of mutant-FLT3 expression by EGCG could be mediated through Hsp90, we confirmed this hypothesis in figure 5A. Base on these confirming data, we explained why EGCG just exerted its inhibition effect on FLT3-ITD but not FLT3-WT. Moreover, we at the first time suggest that others polyphenols including EGC and ECG might also suppressed FLT3 expression through inhibition of Hsp90 function by the same way as EGCG did (Fig. 5B).

One of the effective strategies for treating AML patients with *FLT3* mutations is combination of drugs that have different mechanism of pharmaceutical action. In this study, we combined EGCG with PKC412 to check their combination effect on *FLT3* mutated cell lines. The concentration of both two drugs are significantly reduced (Fig. 7) compared with EGCG alone or PKC412 alone. For examples, the IC_50_ of EGCG alone for MOLM-13 cells was 15 µM, however, when combined with PKC412, the concentration of EGCG will reduce to 10 µM (with 5nM PKC412) and even 5 µM (with 7nM PKC412). Similarly, the IC_50_ of PKC412 alone for MOLM-13 cells was 20nM, however, when combined with EGCG, the concentration of PKC412 will remarkably reduce to 5nM (with 10 µM EGCG). It is important to note that green tea is now developing as a cancer preventive drug in the USA and Europe [23], [24]. It suggests that polyphenols of green tea could be promising candidates for treatment of AML or for combination with other drugs such as FLT3 inhibitors to improve the treatment efficacy.
